# Rapid pathogen detection in synovial fluid of acute native joint infections in adults: a clinical evaluation of a novel automated multiplex polymerase chain reaction (mPCR) system

**DOI:** 10.5194/jbji-10-437-2025

**Published:** 2025-11-05

**Authors:** Lukas Rabitsch, Markus Luger, Felix Lötsch, Peter Starzengruber, Florian Thalhammer, Reinhard Windhager, Birgit Willinger, Irene Katharina Sigmund

**Affiliations:** 1 Department of Orthopaedics and Trauma Surgery, Medical University of Vienna, Währinger Gürtel 18–20, 1090 Vienna, Austria; 2 Division of Clinical Microbiology, Department of Laboratory Medicine, Comprehensive Centre for Infection Medicine, Medical University of Vienna, Währinger Gürtel 18–20, 1090 Vienna, Austria; 3 Department of Urology, Medical University of Vienna, Währinger Gürtel 18–20, 1090 Vienna, Austria

## Abstract

**Aim**: The aim of this study was to investigate the diagnostic performance of a novel rapid multiplex polymerase chain reaction (mPCR) in adults with suspected acute native joint infection. **Methods**: This retrospective single-centre study included 143 patients with suspected acute native joint infection from February 2023 to May 2024. A septic arthritis was classified based on institutional criteria. The agreement between mPCR and conventional culture of synovial fluid (SF) was assessed by calculating the Cohen's κ coefficient. The diagnostic performance of mPCR was calculated, and the area under the curve (AUC) was compared with conventional culture of synovial fluid by using the z test. **Results**: When considering only microorganisms targeted by mPCR, this method detected 13 novel microorganisms in 13 cases compared to conventional culture, resulting in an overall agreement of 91 %, a positive agreement of 100 %, a negative agreement of 88 %, and a Cohen's κ coefficient of 0.780. Of these 13 cases, 9 were classified as septic, with 6 ( n=6/9 , 67 %) on antibiotics prior to aspiration. When considering all microorganisms (including off-panel microorganisms), the overall percentage agreement between mPCR and conventional culture was 89 %, with a Cohen's κ coefficient of 0.735, indicating substantial agreement. Sensitivity, specificity, PPV, NPV, LR + , LR - , accuracy, and AUC of mPCR were 45 %, 89 %, 90 %, 44 %, 4.21, 0.62, 59 %, and 0.671, and those of conventional culture were 40 %, 100 %, 100 %, 45 %, 0.60, 59 %, and 0.698. No difference in performance was observed between both methods ( p=0.183 ). The combination of both techniques showed a sensitivity, specificity, PPV, NPV, LR + , LR - , accuracy, and AUC of 48 %, 89 %, 90 %, 46 %, 4.5, 0.58, 62 %, and 0.686. **Conclusion**: Given its comparable diagnostic performance and faster turnaround time relative to conventional synovial fluid culture, this novel mPCR can be recommended as a valuable adjunct in the diagnosis of septic arthritis in adults, particularly in patients with prior antimicrobial treatment.

## 1 Introduction

Acute native joint infection in adults is a serious condition that can lead to significant joint damage and potentially severe systemic complications if not diagnosed and treated rapidly (Wu et al., 2024). The incidence of septic arthritis varies in the literature, affecting approximately 2–10 per 100 000 individuals annually, with higher rates observed in vulnerable populations such as the elderly and those with predisposing conditions like diabetes, rheumatoid arthritis, or immunosuppression (Wu et al., 2024; Shirtliff and Mader, 2002).

Currently, the diagnosis of acute native joint infections involves a combination of serological, synovial, microbiological, histological, and radiological investigations (Earwood et al., 2021). Serum markers, such as elevated serum C-reactive protein (CRP) and white blood cell (WBC) counts, can be the first indication of septic arthritis, but they lack accuracy (Eren and Aktekin, 2023).

Synovial fluid (SF) analysis often shows elevated leukocyte counts, and a high percentage of polymorphonuclear neutrophils (PMNs) may result in misdiagnosis or delayed initiation of treatment, especially in immunocompromised patients (Aggarwal et al., 2020). Conventional culture of aspirated synovial fluid remains the current gold standard for identifying the causative microorganism. However, this method can be time-consuming and has demonstrated low sensitivity, particularly in patients who have received prior antibiotic therapy (Earwood et al., 2021).

In the last decade, molecular techniques such as polymerase chain reactions (PCRs) have been introduced. They can detect and identify bacterial pathogens based on their genetic characteristics (Sigmund et al., 2019; Achermann et al., 2010; Borde et al., 2015), provide faster results in comparison to traditional culture methods, and detect difficult-to-culture pathogens (Aggarwal et al., 2020). In various orthopaedic settings, multiplex PCRs (mPCRs) have shown similar performances compared to conventional culture (Achermann et al., 2010; Portillo et al., 2012; Sigmund et al., 2018, 2019). For instance, high agreements between these two methods have been described for diagnosing periprosthetic joint infections (PJIs) (Vasoo et al., 2015; Berinson et al., 2023).

In recent years, a novel, fully automated mPCR (BioFire^®^ Joint Infection Panel (BF-JIP), bioMérieux, Marcy-l'Étoile, France) has been designed to identify 31 clinically relevant pathogens and 8 antimicrobial resistance genes directly from synovial fluid within approximately 1 h. Some studies already evaluated its performance in musculoskeletal infections (Salar-Vidal et al., 2023; Esteban et al., 2023; Pascual et al., 2024). However, its clinical efficacy in diagnosing only acute native joint infections remains unclear. Hence, the aim of this study was to evaluate the performance of the novel automated multiplex PCR system in diagnosing acute native joint infections in adults. Additionally, a comparison between mPCR and conventional culture was performed.

## 2 Material and methods

### 2.1 Study design

This retrospective single-centre study was performed between February 2023 and May 2024 at a tertiary healthcare centre specialized in treating musculoskeletal infections. Ethical approval was obtained from the institutional ethical review board (EK 2172/2023) and was conducted in accordance with the Declaration of Helsinki. Adult patients ( ≥18 years) with a red, swollen, and painful joint suspected of having an acute native joint infection were eligible for inclusion. All patients who presented with a joint effusion and a strong clinical suspicion of septic arthritis during the study period and who underwent synovial fluid aspiration were considered for inclusion. Patients were identified by searching the institutional electronic medical records system using relevant diagnostic codes and clinical documentation. The decision to perform mPCR testing was made at the discretion of the treating surgeon and was not uniformly applied to all cases during the study period. Only patients for whom mPCR testing was performed were included in the analysis.

### 2.2 Definitions

Acute native joint infection was defined based on our institutional criteria (Table 1). A joint was defined as septic when one of the following criteria was fulfilled: (i) a draining sinus from the joint, (ii) a synovial fluid leukocyte count of >50000 G/L, (iii) >90 % polymorphonuclear neutrophils (PMN %) in the synovial fluid, (iv) detection of a high-virulence microorganism in the synovial fluid, (v) ≥2 positive cultures with phenotypically identical microorganisms from an affected joint (synovial fluid, deep tissue culture), (vi) the presence of ≥5 neutrophils in ≥5 high-power field (HPF) in histopathological samples, and/or (vii) the presence of visible microorganisms in the histological examination.

**Table 1:** Institutional criteria for the diagnosis of clinically suspected septic arthritis.

	Infection confirmed
	(at least one positive criterion)
**Clinical workup**	
Clinical features	Draining sinus from joint
**Synovial fluid cytological analysis**	
Leukocyte count (G/L)	>50000
PMN (%)	>90 %
**Microbiology**	
Aspiration fluid	Positive culture of a highly virulent microorganism
Intraoperative (fluid and tissue)	≥2 positive samples with the same microorganism
**Histology**	
High-power field (400 × magnification)	Presence of ≥5 neutrophils in ≥5 HPF
	Presence of visible microorganisms

White blood cell count (WBC), percentage of polymorphonuclear neutrophils (PMN %), high-power field (HPF).

### 2.3 Automated multiplex PCR

For the molecular detection of the causing microorganism, a novel automated multiplex PCR system (BioFire^®^ Joint Infection Panel (BF-JIP), bioMérieux, Marcy-l'Étoile, France) was used. This system is able to detect 29 bacterial species, 2 fungal pathogens, and 8 antimicrobial resistance genes directly from synovial fluid specimens. The panel was used according to the manufacturer's instructions and guidelines for processing each synovial fluid sample (200 µ L). This test involves initial sample lysis and nucleic acid extraction and purification, followed by nested multiplex PCR amplification. Results are then generated based on endpoint melting curve analysis.

### 2.4 Conventional culture of synovial fluid

Synovial fluid samples were cultured using different media to ensure comprehensive pathogen detection. For aerobic cultures, BD Columbia agar III with 5 % sheep blood (Becton Dickinson GmbH, Heidelberg, Germany) with an *S. aureus* satellite nurse strain was incubated at 35–37 °C in a CO_2_-enriched atmosphere for up to 2 weeks. Additionally, MacConkey agar No. 3 (Thermo Fisher Scientific, Oxoid Ltd, Basingstoke, UK) was used for aerobic culturing over a 48 h period, and brain heart infusion medium supplemented with 0.1 % agar (Thermo Fisher Scientific, Oxoid Ltd, Basingstoke, UK) was inoculated and incubated for up to 2 weeks. Anaerobic cultures were performed using Brucella blood agar enriched with haemin and vitamin K1 (Becton Dickinsin GmbH, Heidelberg, Germany), along with Schaedler kanamycin–vancomycin agar containing 5 % sheep blood (Becton Dickinson GmbH, Heidelberg, Germany), with incubation extending to 2 weeks. For fungal culture, BBL^TM^ CHROMagar^TM^ Candida Medium (Becton Dickinson GmbH, Heidelberg, Germany), Sabouraud dextrose (SAB) agar (Becton Dickinson GmbH, Heidelberg, Germany), SAB brain heart infusion (BHI) slant agar (Becton Dickinson GmbH, Heidelberg, Germany), and SAB Bouillon (bioMérieux SA, Marcy-l'Étoile, France) were inoculated. Antimicrobial susceptibility testing was performed in accordance with the European Committee on Antimicrobial Susceptibility Testing (EUCAST) guidelines using the disc diffusion method (EUCAST, 2025).

### 2.5 Statistical analysis

Categorical variables were expressed as absolute frequencies ( n ) and percentages (%), while continuous variables were presented as median with interquartile range (IQR) depending on their distribution. To assess the agreement between mPCR and conventional culture, positive, negative, and overall percentage agreement were calculated, along with Cohen's κ coefficient. Additionally, sensitivity, specificity, positive and negative likelihood ratios (LR + and LR - ), and predictive values (positive and negative) of mPCR, conventional culture, and both diagnostic methods combined were calculated against our institutional criteria. The discriminative ability of each test was quantified by the area under the receiver operating characteristic (ROC) curve (AUC). Statistical analyses were performed using XLSTAT (version 2023.3.1). For all tests, a p value of less than 0.05 was considered statistically significant.

**Figure 1:**
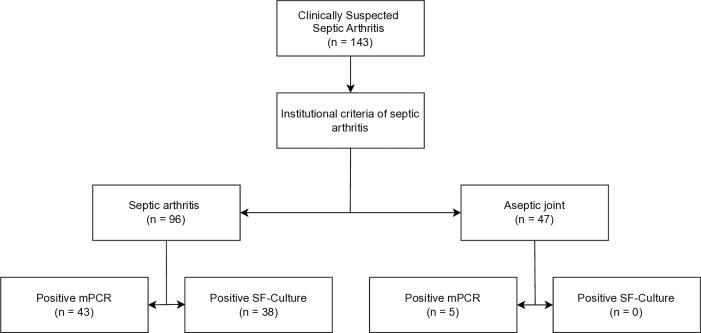
Flowchart of all included patients with a clinical suspicion of septic arthritis.

**Table 2:** Demographic data of all patients with and without septic arthritis, according to our institutional criteria of septic arthritis.

Demographic data	Septic joint	Aseptic joint	p value	All patients
	( n=96 )	( n=47 )		( n=143 )
Age (years, median, IQR)	62 (48–75)	61 (38–77)	0.354^a^	62 (46–76)
Female patients, ( n , %)	34 (35)	15 (32)	0.679^b^	49 (34)
Rheumatoid arthritis ( n , %)	4 (4)	5 (11)	0.258^b^	9 (6)
Antibiotics prior to aspiration ( n , %)	37 (39)	14 (30)	0.305^b^	51 (36)
ASA (median, IQR)	3.0 (2–3)	2.0 (2–3)	$<0.0001^{a,d}$	2.5 (2–3)
BMI (median, IQR)	24.5 (23–28)	26.6 (24–30)	$<0.0001^{a,d}$	24.7 (23–29)
Joint, n (%)				
Knee	69 (72)	42 (89)	$0.018^{b,d}$	111 (78)
Hip	5 (5)	2 (4)	0.869^c^	7 (5)
Shoulder	15 (16)	0 (0)	$0.010^{c,d}$	15 (10)
Elbow	5 (5)	0 (0)	0.268^c^	5 (3)
Upper ankle joint	2 (2)	3 (6)	0.406^c^	5 (3)

^a^ Mann–Whitney test. ^b^ $\chi^{2}$ test. ^c^ Fisher's exact test. ^d^ Statistically significant.

## 3 Results

A total of 179 patients with suspected acute native joint infection were identified, of whom 143 underwent mPCR testing and were included in the analysis. Of these, 96 patients (67 %) were diagnosed with septic arthritis, and the remaining 47 (43%) patients were classified as aseptic (Fig. 1). No difference was observed between both groups regarding age, gender, and rheumatoid arthritis (Table 2). Patients in the septic group had a statistically significantly higher ASA score and BMI ( p<0.0001 ) and received antibiotics more often prior to aspiration ( p<0.0001 ). Knees were affected in the majority of patients (78 %, n=111/143 ), followed by shoulders (10 %, n=15/143 ), hips (5 %, n=7/143 ), elbows (3 %, n=5/143 ), and upper ankle joints (3 %, n=5/143 ). mPCR identified a total of 48 microorganisms in 48 synovial fluid samples, while conventional SF culture detected 41 microorganisms (Table 3) in 38 SF samples. *S. aureus* was the most common pathogen detected by both methods (Table 3), followed by *Streptococcus* spp. and *E. coli*. In comparison to conventional culture, mPCR was able to detect *S. aureus* in four additional synovial fluid samples, *Streptococcus* spp. in six samples, *E. coli* in one sample, and *S. lugdunensis* in one additional sample (Table 3).

**Table 3:** Distribution of microorganisms detected by multiplex PCR and in the synovial fluid (SF) culture with and without antibiotics.

Isolated microorganism,	mPCR	Conventional	mPCR with	Conventional
n (%)	( n=48)	culture	antibiotics	culture with
		( n=41 )	( n=23 )	antibiotics
				( n=16 )
***Staphylococcus aureus***	25 (52.1)	21 (51.2)	13 (56.5)	11 (68.8)
***Staphylococcus lugdunensis***	1 (2.1)	0 (0)	0 (0)	0 (0)
***Streptococcus* spp.**	16 (33.3)	10 (24.4)	7 (30.4)	3 (18.8)
**Group A streptococci**	0	3	0	2
**Group B streptococci**	0	3	0	0
**Group G streptococci**	0	2	0	0
***Streptococcus agalactiae***	3	1	2	1
***Streptococcus pyogenes***	3	0	1	0
***Streptococcus mitis/oralis***	0	1	0	0
***Escherichia coli***	5 (10.4)	4 (9.8)	3 (13.0)	2 (12.5)
***Serratia marcescens***	1 (2.1)	1 (2.4)	0 (0)	0 (0)
*Staphylococcus hominis*	0 (0)	1 (2.4)	0 (0)	0 (0)
*Staphylococcus epidermidis*	0(0)	3 (7.3)	0 (0)	0 (0)
*Acinetobacter ursingii*	0 (0)	1 (2.4)	0 (0)	0 (0)
Polymicrobial	0 (0)	3 (7.3)	0 (0)	3 (18.8)
Total	48 (100)	41 (100)	23 (100)	16 (100)

Note: microorganisms included in the multiplex PCR (mPCR) panel are highlighted in bold. A thick line separates on-panel organisms from off-panel organisms.

### 3.1 Overall percentage agreement and discrepancies between the two methods

The overall percentage agreement between mPCR and conventional synovial fluid culture was 89 % (95 % CI, 84–94) with a Cohen's κ coefficient of 0.735, indicating substantial agreement. When only evaluating microorganisms targeted by mPCR, the overall agreement and Cohen's κ coefficient were calculated to be slightly higher: 91 % (95 % CI: 86–96) and 0.780. Positive agreement was 92 % (95 % CI: 78–98) for all microorganisms and 100 % (95 % CI: 88–100) for microorganisms targeted by mPCR. Negative agreement was 88 % (95 % CI: 80–93) both for all microorganisms and for microorganisms targeted by mPCR (Table 4).

**Table 4:** Percentage agreement between mPCR and synovial fluid culture.

Statistic	Agreement, all	Agreement,
	microorganisms	microorganisms
	( + off panel MO)	targeted by mPCR
Overall % agreement (95 % CI)	88.8 % (83.6–94.0)	90.7 % (85.9–95.5)
Positive % agreement (95 % CI)	92.1 % (78.3–97.9)	100 % (87.9–100)
Negative % agreement (95 % CI)	87.6 % (79.8–92.7)	87.6 % (79.8–100)
Cohen's κ coefficient	0.735 (0.613–0.857)	0.780 (0.666–0.894)

CI: confidence interval; MO: microorganism(s).

In three cases, conventional culture identified microorganisms that were not covered by the mPCR panel (Table 5): *Staphylococcus hominis* grew in one patient with a painful, swollen, and red right knee, fever (38.5 °C), and an SF-WBC of 55 450 G/L. In a 28-year-old patient with psoriatic arthritis, Crohn's disease and perianal fistula, immunosuppressive therapy, and history of a previous septic arthritis of both knees, synovial fluid cultures showed growth of *Acinetobacter ursingii*. *Staphylococcus epidermidis* was cultured in one 77-year-old patient with a painful, swollen, and red right elbow and a recently diagnosed acute myeloid leukaemia. Serum CRP was 12 mg dL^−1^, WBC was 1.6 G/L, synovial fluid WBC was 5330 G/L, and % PMN was 92 %. All of these latter three microorganisms are not included in the mPCR panel.

**Table 5:** Culture-positive cases with pathogens not targeted by the mPCR assay.

Patient	Gender	Age	Site	Abx	Septic	mPCR	Culture	Serum CRP	SF-WBC	SF-	Histology	Tissue
								(mg dL^−1^)	(G/L)	% PMN		culture
1	M	73	Knee	N	Septic	Neg.	*Staph. hominis*	6.75	55 450	77	NA	NA
2	F	28	Knee	N	Septic	Neg.	*Acinetobacter ursingii*	8.77	31 730	83	NA	NA
3	M	77	Elbow	N	Septic	Neg.	*Staph. epidermidis*	11.98	5330	92	NA	NA

CRP: C-reactive protein; PMN: polymorphonuclear neutrophils; *Staph. hominis*: *Staphylococcus hominis*; *Staph. epidermidis*: *Staphylococcus epidermidis*. NA: not available.

In 13 cases, mPCR identified pathogens, while conventional culture was negative (Table 6): of these, nine cases (69 %) were classified as septic based on at least one fulfilled criterion (SF-WBC > 50 000 G/L, SF-PMN % > 90 %, positive histology, and/or positive tissue culture; Table 6). It is noteworthy that six out of these nine septic cases (67 %) were on antibiotics prior to aspiration. Overall, a false positive result was observed in four cases (patients 3, 6, 10, and 12 in Table 6).

**Table 6:** Cases with negative synovial fluid culture and positive mPCR results.

Patient	Gender	Age	Site	Abx	Septic	mPCR	SF	Serum CRP	SF-WBC	SF-	Histology	Tissue	
							culture	(mg dL^−1^)	(G/L)	% PMN		culture	
1	M	68	Knee	Y	Septic	*Streptococcus* spp.	Neg.	25.5	183 300	81	Infection	NA	
2	M	68	Knee	Y	Septic	*Streptococcus* spp.	Neg.	25.3	44 640	97	Infection	NA	
3	F	25	Knee	N	Aseptic	*Staph. lugdunensis*	Neg.	0.04	6859	38	NA	NA	
4	M	76	Knee	Y	Septic	*Staph. aureus*	Neg.	14.91	78 410	97	NA	NA	
5	M	63	Knee	N	Septic	*Staph. aureus*	Neg.	19.96	296 900	96	Infection	*Citrobacter freundii*,	
												*Staph. aureus*,	
												*Enterococcus faecalis*,	
												*Streptococcus anginosus*	
6	M	62	Knee	N	Aseptic	*Staph. aureus*	Neg.	NA	9551	NA	NA	NA	
7	M	81	Knee	N	Septic	*Streptococcus* spp.	Neg.	26.0	36 055	97	NA	NA	
8	M	35	Knee	N	Septic	*Streptococcus* spp.	Neg.	25.46	34 290	95	NA	NA	
9	F	55	Ankle	Y	Aseptic	*Staph. aureus*	Neg.	4.98	NA	NA	NA	*Staph. aureus*	
10	M	27	Knee	N	Aseptic	*Serratia marcescens*	Neg.	12.78	<1	NA	NA	NA	
11	M	46	Hip	Y	Septic	*Streptococcus* spp.	Neg.	5.4	NA	NA	Infection	NA	
12	M	82	Knee	Y	Aseptic	*Escherichia coli*	Neg.	15.9	28 920	77	NA	NA	
13	F	82	Shoulder	Y	Septic	*Streptococcus* spp.	Neg.	11.07	NA	NA	Infection	*Staph. epidermidis*	

CRP: C-reactive protein; PMN: polymorphonuclear neutrophils; *Staph. aureus*: *Staphylococcus aureus*; *Staph. epidermidis*: *Staphylococcus epidermidis*; *Staph. lugdunensis*: *Staphylococcus lugdunensis*; *Streptococcus* spp.: *Streptococcus* species; SF: synovial fluid; NA: not available.

**Figure 2:**
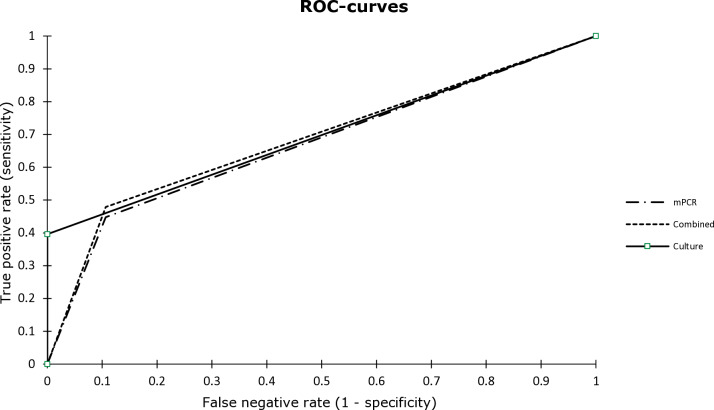
Receiver operating characteristic (ROC) curves illustrating accuracies of mPCR, synovial fluid (SF) culture, and combined evaluation (SF culture + mPCR). No statistically significant difference was observed between the performances of mPCR and conventional culture ( p=0.183 , z test).

### 3.2 Performance of the novel automated multiplex PCR

Considering all cases, the sensitivity, specificity, PPV, NPV, LR + , LR - , accuracy, and AUC of mPCR were 45 %, 89 %, 90 %, 44 %, 4.21, 0.62, 59 %, and 0.671, respectively (Table 7). Although a higher sensitivity was observed (45 %, 95 % CI: 35–55) in comparison to conventional culture (40 %, 95 % CI: 30–50), mPCR demonstrated a lower specificity (89 %, 95 % CI: 77–96 vs. 100 %, 95 % CI: 100). No statistically significant difference between the performances of both test methods was seen ( p=0.183 , z test; Fig. 2). When combining mPCR and conventional culture, a higher sensitivity of 48 % was calculated with a comparably high AUC compared to conventional culture (Table 7). When considering only patients receiving antimicrobial therapy, the sensitivity of mPCR increased to 56.8 % (95 % CI: 40.9–71.3), while specificity slightly decreased to 85.7 % (95 % CI: 58.6–97.0), and the AUC slightly increased to 0.712 (95 % CI: 0.587–0.837).

**Table 7:** Performance of mPCR, synovial fluid (SF) culture, and combined results for the diagnosis of septic arthritis.

Performance parameter	mPCR	SF culture	Combined
	(95 % CI)	(95 % CI)	(95 % CI)
Sensitivity, %	44.8 (35.2–54.8)	39.6 (30.4–49.6)	47.9 (38.2–57.8)
Specificity, %	89.4 (76.8–95.7)	100 (90.8–100)	89.4 (76.8–95.7)
Accuracy, %	59.4 (51.4–67.5)	59.4 (51.4–67.5)	61.5 (53.6–69.5)
Positive predictive value, %	89.6 (80.9–98.2)	100 (100)	90.2 (82.0–98.4)
Negative predictive value, %	44.2 (34.2–54.2)	44.8 (35.3–54.3)	45.7 (35.5–55-8)
Positive likelihood ratio	4.21 (1.79–9.93)	–	4.50 (1.92–10.59)
Negative likelihood ratio	0.62 (0.50–0.76)	0.60 (0.51–0.71)	0.58 (0.47–0.72)
Area under the curve	0.671 (0.604–0.738)	0.698 (0.649–0.747)	0.686 (0.619–0.754)

CI: confidence interval; SF: synovial fluid.

## 4 Discussion

In our cohort, a substantial agreement between the mPCR and conventional culture of synovial fluid samples was observed when diagnosing native septic arthritis in adults. When only microorganisms targeted by mPCR were considered, the overall agreement was 91 % with a Cohen's κ coefficient of 0.78. Similar results have been described in a retrospective multi-centre study of 1544 synovial fluid samples with a positive agreement of 91 % and a negative agreement of 100 % (Esteban et al., 2023). However, samples from patients with septic arthritis, periprosthetic joint infections (PJIs), and an infection of unknown origin have been evaluated combined without further sub-analysis of the different musculoskeletal infection types. It is known that low-virulence microorganisms, such as coagulase-negative staphylococci, are the most common causing organisms in chronic PJI (Luger et al., 2024). However, no target gene for these microorganisms is included in this novel mPCR panel. Hence, their results cannot be extrapolated to patients with native septic arthritis alone, in whom these organisms are rather uncommon. A more detailed and separated analysis, particularly of the different musculoskeletal infection types, would be reasonable. Furthermore, no statistical analysis including off-panel organisms has been performed.

When all microorganisms (including off-label pathogens) were considered in our cohort, the overall agreement was 89 % with a Cohen's κ coefficient of 0.74. The positive agreement was 92 %, and the negative agreement was 88 %. In comparison, a lower positive agreement of 69 % between mPCR and conventional culture has been reported in a prospective, multi-centre study of 262 synovial fluid samples when including off-panel pathogens, while the negative agreement has been similar (92 %) (Salar-Vidal et al., 2023). However, patients with septic arthritis, PJI, and osteomyelitis combined have again been analysed without further differentiation between musculoskeletal infection types. As mentioned above, a more detailed investigation would be beneficial.

In another retrospective multi-centre study including 1527 samples from patients with septic arthritis ( n=873 ), PJI ( n=398 ), and unknown origin ( n=185 ), a sub-analysis has been performed (Pascual et al., 2024). Cases with native septic arthritis have shown an overall agreement of 88 %, which is in line with our findings. However, performance analysis for both test methods (mPCR, conventional culture) evaluating their value in clinical routine has not been performed. This present study is the first to report the diagnostic accuracy of mPCR and conventional culture. Our results showed similar performances of both test methods with a higher identification rate of microorganisms in patients receiving antimicrobial therapy prior to aspiration when using mPCR. In this subgroup, mPCR demonstrated improved diagnostic performance, reflected by increased sensitivity and overall accuracy. These findings align with those from previous studies emphasizing the clinical utility of PCR-based diagnostics in cases where traditional culture methods are compromised by antimicrobial exposure. However, as with other PCR-based assays, the mPCR may occasionally accept a low specificity for enhanced sensitivity, a limitation attributed to the potential detection of non-viable organisms or contaminants (Goswami et al., 2022; Sigmund et al., 2019; Achermann et al., 2010). Despite the improved sensitivity observed in patients with prior antibiotic exposure, the clinical interpretation of mPCR results must still account for the possibility of false positives and their implications for patient management. Although the false positive rate of mPCR was low in our cohort ( n=4 ), results should be interpreted with caution. A positive mPCR result in the absence of supporting clinical or laboratory findings should be regarded as potential contamination rather than a true infection. In contrast, a positive result in patients who have received prior antimicrobial therapy (particularly those with borderline synovial fluid findings, which may be influenced by prior antibiotics) should raise a high index of suspicion for the identified microorganism being the true causative pathogen.

Compared to alternative PCR technologies, this novel mPCR offers distinct advantages, including its rapid turnaround time of approximately 1 h and its broad pathogen coverage. Unlike single-target assays or systems requiring complex pre-analytical steps, this novel mPCR efficiently identifies multiple pathogens and several resistance genes simultaneously (Borde et al., 2015; Esteban and Gómez-Barrena, 2021). While other mPCR platforms may include a wider variety of organisms, they often require longer processing times, potentially limiting their practical use in emergency settings (Borde et al., 2015; Hischebeth et al., 2016). Moreover, other systems have faced criticism for their lack of sensitivity in scenarios involving low bacterial loads (Malandain et al., 2018). Given that septic arthritis demands urgent intervention, this novel mPCR may allow earlier targeted antimicrobial therapy and potentially improved patient outcomes. However, while rapid identification of pathogens through mPCR testing is an attractive concept, its impact on immediate clinical management in acute native joint infections may be limited. In our institution, surgical intervention is typically initiated based on clinical presentation and synovial fluid analysis (SF-WBC > 50 000 G/L, SF-% PMN > 90 %), regardless of microbiological confirmation. Empiric antibiotic therapy is generally sufficient to cover the pathogens later identified by conventional culture or mPCR. As such, earlier organism identification would not have significantly altered the timing of surgery or initial antibiotic choice in most cases. Nevertheless, mPCR may still offer clinical value in selected situations, such as in patients already receiving antimicrobial therapy, in those with atypical clinical presentations or known antibiotic allergies, or in cases where rapid de-escalation of broad-spectrum antibiotics is desirable.

Furthermore, mPCRs are not intended as standalone tests to identify microorganisms. Conventional culture remains indispensable for generating antimicrobial susceptibility profiles and guiding long-term treatment strategies. In addition, such mPCRs are limited by their included target genes. In the case of the investigated mPCR, other important microorganisms, such as coagulase-negative staphylococci, *Cutibacterium acnes*, and *Corynebacterium* spp., cannot be detected, restricting its applicability in some clinical scenarios (Tsikopoulos and Meroni, 2023; Tan et al., 2018; Borde et al., 2015). However, these microorganisms rarely cause septic arthritis and are more common in cases of chronic PJI.

This study has several limitations: the retrospective design and single-centre setting may limit the generalizability of the findings to other healthcare systems. The sample size may have constrained the statistical power and limited our ability to identify subtle differences in performance. Not all parameters of our institutional infection definition were available to all patients, which is a common issue in clinical routine. Future prospective, multi-centre studies with larger cohorts are warranted to validate these findings and to further assess the clinical utility and cost-effectiveness of this novel mPCR in diagnosing acute native joint infections.

## 5 Conclusion

With a similar performance in comparison to conventional culture, this novel mPCR represents a valuable adjunct in the diagnosis of septic arthritis in adults, particularly in patients with prior antimicrobial treatment. Its rapid turnaround time and comparable diagnostic accuracy may enhance the clinician's ability to initiate timely, targeted antimicrobial therapy. However, its limitations underscore the need for complementary use with culture-based methods to ensure comprehensive pathogen identification and antimicrobial susceptibility testing.

## Data Availability

All data generated or analysed during this study are included in this published article. The main datasets consist of the data presented in Tables 1–7 and Figs. 1–2.
